# Experimental Validation of Simple Power Quality Indices for Frequency Content Assessment up to 150 kHz

**DOI:** 10.3390/s25216716

**Published:** 2025-11-03

**Authors:** Christian Betti, Roberto Tinarelli, Lorenzo Peretto, Alessandro Mingotti

**Affiliations:** Department of Electrical, Electronic and Information Engineering, Guglielmo Marconi Alma Mater Studiorum, University of Bologna, Viale del Risorgimento 2, 40136 Bologna, Italy; christian.betti2@unibo.it (C.B.); roberto.tinarelli3@unibo.it (R.T.); lorenzo.peretto@unibo.it (L.P.)

**Keywords:** power quality, instrument transformers, supraharmonics, indices, high frequency, harmonic distortion

## Abstract

The power system is evolving with the integration of new technologies, including electronic devices and renewable energy sources, which are increasingly used to support new applications, reduce dependence on fossil fuels, and drive system innovation. However, this shift brings a significant drawback: a reduction in power quality (PQ). The literature extensively discusses the impact of poor PQ on electrical assets and explores potential solutions to this new challenge. Building on this foundation, this paper introduces new PQ indices derived from existing metrics and validated on both synthetic and real signals to assess their effectiveness. The aim is to provide researchers and system operators with simple and efficient tools for the clear identification of PQ issues in monitored networks. These new indices are designed to be flexible and independent of acquisition conditions, making them suitable for a wide range of frequencies (e.g., 50 Hz–150 kHz) and applications. After an overview of the PQ landscape, the paper demonstrates the use of these indices on various voltage waveforms, including a case study from a measurement campaign. The promising results indicate that, when combined with existing indices, these new metrics can form a strong foundation for a deeper understanding and more accurate classification of PQ issues in power networks.

## 1. Introduction

The integration of renewable energy sources (RESs) and the proliferation of electronic devices (EDs) are having a significant impact on power quality (PQ) in modern power systems [1]. RESs, such as solar photovoltaics (PV) and wind turbines, use switching power electronics—inverters—to convert direct current (DC) to alternating current (AC). Although these devices are essential to efficiently connect the RESs to the grid, they also introduce harmonic distortions across a wide frequency spectrum. As inverters operate in discrete pulses, they cause harmonic current injections across a wide frequency spectrum [2]. These distortions lead to non-sinusoidal voltage and current waveforms, especially in the mid-to-high-frequency ranges, such as from 9 kHz to 150 kHz [3]. Additionally, the widespread use of EDs in households, commercial buildings, and industries also contributes to PQ issues. In fact, many modern devices, such as LED lighting, computers, and electric vehicles [4], incorporate switching power supplies and other electronic components that draw non-linear current from the grid. These devices generate harmonics and other disturbances in both low- and high-frequency ranges [5]. Together, the non-linear nature of RESs and EDs introduces various disturbances, including harmonic distortion, flicker, and voltage fluctuations. These distortion phenomena create non-ideal operating conditions, leading to physical impacts on electrical assets such as overheating, vibration, and insulation degradation in motors, transformers, and cables [6,7]. Moreover, sensitive equipment, such as control systems, medical devices, and data servers, is especially vulnerable to poor PQ. Voltage sags, surges, and flicker can cause frequent shutdowns, reduce operational stability, and lead to data losses or inaccurate measurements [8]. Ultimately, low levels of PQ impact the efficiency, longevity, and reliability of power distribution; consequently, economic implications arise, such as higher system maintenance costs and fines and economic losses in case of power outages [9]. Consequently, due to the accelerating trend toward RESs, distributed generation, and EDs, developing new indices to evaluate these PQ issues across all frequency ranges has become a crucial task for system operators in order to ensure a stable and resilient power system for modern needs.

Overall, this work aims to introduce simple PQ indices designed to assess the spectral content of a signal, selectively focusing on specific portions of the spectrum. These indices are demonstrated primarily in the 50 Hz–150 kHz frequency range but are not limited to it. The indices are of straightforward implementation, and they provide an immediate indication of the signal’s quality and “health”, specifying in which frequency range the criticalities are found. Of course, several PQ indices are defined in the literature and in established international standards, all addressing specific needs and providing a robust framework for harmonic analysis (see the dedicated section in what follows). However, the added value of the proposed indices, hence of the work, is to have a simple and practical diagnostic tool for an immediate evaluation of the signal spectral content and, in particular, the identification of the certain frequency band in which the distortion is most prominent.

Furthermore, after describing them, the indices are tested in two different conditions. First, a validation in a simulation environment with synthetic signals is run. Such signals are representatives of potential voltages and current measured in the power system. Second, the indices are tested on signals obtained within a measurement campaign aimed at acquiring voltages signals generated by a PV system. Both testing procedures confirm the flexibility, reliability, and ease of use of the proposed PQ indices. Note that the proposed indices could, in principle, be tested across thousands of scenarios involving different mixtures of harmonic and frequency components, as they are flexible and applicable to all such cases. However, their validation has been presented using a single case study, which is considered sufficiently general and robust to demonstrate their effectiveness.

The remainder of the text is structured as follows: Section 2 outlines the context of this paper, analyzing the existing indices and standards. Section 3 introduces the proposed indices and their meaning. In Section 4, the indices are tested in the simulation environment. The experimental validation, instead, is fully described in Section 5. Finally, Section 6 contains the conclusion of the work.

## 2. State of the Art

This section starts with the analysis of the existing and typical PQ indices. Afterwards, a brief discussion on instrument transformers (ITs) is provided to clarify their crucial role.

### 2.1. Power Quality Evaluation

#### 2.1.1. Standards

Many standards on PQ are available. Some of them prescribe tests, some device specifications and limits, and others focus on the user or manufacturer perspective. This review focuses specifically on the relevant standards that define measurement methods and indices for quantifying waveform distortion to provide a context in which to compare the indices proposed by the authors. Among them, some of the most popular are the IEC 61000-4-7 [10] and the IEC 61000-4-30 [11], in which the total harmonic distortion (THD) is used together with grouping technique introduced. The THD is defined as

(1) $$THD=\frac{\sqrt{\sum_{h=2}^{h\_max}Y_{H,h}^{2}}}{Y_{H,1}}$$

where $Y_{H,h}$ is the rms value of the considered quantity Y at the harmonic component of order h. $Y_{H,1}$ is the rms value of the fundamental component. In [10], the partial weighted harmonic distortion (PWHD) is also introduced, which is a weighted version of the THD. Another document indicating an index is the IEC 63191 [12], in which the total distortion ratio (TDR), also known as total waveform distortion (TWD), and the total demand distortion are used (TDD). They are defined as

(2) $$TWD=\frac{\sqrt{Y_{H}^{2}-Y_{H,1}^{2}}}{Y_{H,1}}$$

(3) $$TDD=\frac{\sqrt{\sum_{h=2}^{h\_max}Y_{H,h}^{2}}}{Y_{H,1,max}}$$

where $Y_{H}$ is the overall rms of the quantity Y, and $Y_{H,1,max}$ is the max demand value. Then, the IEEE 519 [13] sets some limits for the harmonic components of the current, but the only index used is the telephone interference factor (*TIF*), whose use is limited to the communication assessment. Finally, ref. [14] provides comprehensive and detailed guidance on a wide range of voltage sag indices, ranging from single event characteristics (such as sag energy) to site indices (such as SARFI-X), while ref. [15] offers indices to measure the severity of the flicker in the short and long term, $P_{st}$ and $P_{lt}$, respectively.

Overall, the guidelines of the standards provide an excellent starting point for the PQ evaluation, but there is plenty of space for improvements.

#### 2.1.2. Literature Review

Studying the state of the art, some examples of new interesting indices can be found. For example, ref. [16] introduced a sort of ratio error using the THD values. In [17], the PQ evaluation approach is based on the traffic light technique. The authors in [18] introduce a distortion index after applying a wavelet-based approach. In [19], a sort of review of some indices is given (all of which have been already mentioned). The same concept has been applied in [20], where the shortcomings of the existing indices have been highlighted. As for [21], the proposed solution is specific for evaluating the impact of photovoltaic sources and non-linear loads. Meanwhile, ref. [22] focuses on the proposal of indices that can be used for characterizing the behavior of electric drives. Then, ref. [23] proposes novel PQ indices that, by means of power decomposition method, assess PQ phenomena in case of using LED lamps. Finally, ref. [24] introduces a short time version of the THD, the STDE, which quantifies the distortion by considering the energy of all frequency components. Considering the above, what is proposed following this section clearly represents the value this adds for the scientific community.

### 2.2. Instrument Transformers

This paragraph aims to emphasize the importance of ITs in PQ evaluation. In fact, any PQ index would be useless if the input provided is inaccurate or even wrong. Hence, considering that ITs are the key link between the electrical quantities and the computation of parameters/indices, they have a fundamental role. Traditional ITs are generally designed for fundamental frequency measurements (50 or 60 Hz) and may fail to accurately capture the higher-frequency components introduced by modern loads. This gap in measurement capabilities can result in inaccurate monitoring, inadequate control responses, and a limited understanding of PQ issues in high-frequency ranges, such as from 9 kHz to 150 kHz. Therefore, there is a pressing need for specialized ITs capable of measuring and analyzing these frequency components to enable accurate PQ assessment. This need is confirmed by the latest version of the main IT standard document, the IEC 61869-1 [25], and the most recent literature [26,27], as well as the financing of two European projects, IT4PQ (19NRM05) [28] and ADMIT (22NRM06) [29], which aimed to study the frequency response of ITs up to 9 kHz and 150 kHz, respectively.

## 3. Proposed Indices

This section presents the indices and the metrics developed as a simple and immediate diagnostic tool for evaluating the harmonics that are affecting the system under analysis. The standard THD calculation provides a global measure of waveform distortion but fails to isolate the contribution of different spectral regions. Therefore, to overcome this limitation, this work introduces a set of modular indices designed to quantify the aggregated distortion energy within specific and predefined frequency bands. By calculating a THD value for distinct spectral ranges, a system operator can quickly determine whether the dominant PQ issues originate from classic low-order harmonics, high-frequency switching components, or other sources. The first set of three indices includes ${THD}_{PQ}$, ${THD}_{EX}$, and ${THD}_{SH}$. They are defined as

(4) $${THD}_{PQ}=\frac{\sqrt{\sum_{h=2}^{50}Y_{H,h}^{2}}}{Y_{H,1}}$$

(5) $${THD}_{EX}=\frac{\sqrt{\sum_{h=51}^{180}Y_{H,h}^{2}}}{Y_{H,1}}$$

(6) $${THD}_{SH}=\frac{\sqrt{\sum_{h=181}^{450}Y_{H,h}^{2}}}{Y_{H,1}}$$

where the subscripts PQ, EX, and SH refer to power quality, extended PQ, and supraharmonics. In fact, the three equations introduce a sort of THD dedicated to a specific part of the frequency spectrum, as outlined in [10,11]. Hence, ${THD}_{PQ}$ evaluates harmonic components up to 2500 Hz; ${THD}_{EX}$ considers the frequency range [2550–9000 Hz], and ${THD}_{SH}$ is specific for the frequencies [9050–150,000 Hz]. Equations (4)–(6) are written in a general form that indicates h in the sum as the harmonic order considered. However,

- The 50 Hz resolution is typically not adopted by the standard [10], and it does not allow us to appreciate interharmonic components.
- In this paper, the analysis at a 50 Hz resolution is integrated with other resolutions to further support the results. In fact, increasing the resolution allows us to introduce the fourth index as

(7) $${THD}_{SUB}=\frac{\sqrt{\sum_{h=1/10}^{9/10}Y_{H,h}^{2}}}{Y_{H,1}}$$

where subscript SUB refers to the subharmonic components from the DC to the fundamental one (both excluded). The superscript and subscript of the summation reflect the case of 5 Hz resolution (for example). In conclusion, the relevant information preserved is therefore the aggregated harmonic distortion for each frequency band under analysis. In this work, the indices quantify and isolate the aggregated distortion within the previously defined frequency bands (PQ, EX, SH, SUB).

To further support this approach, new metrics are defined starting from the previous indices. Such metrics are

(8) $$\rho_{PQ}=\frac{{THD}_{PQ}}{TWD}$$

(9) $$\rho_{EX}=\frac{{THD}_{EX}}{TWD}$$

(10) $$\rho_{SH}=\frac{{THD}_{SH}}{TWD}$$

(11) $$\rho_{SUB}=\frac{{THD}_{SUB}}{TWD}$$

in which all the elements were previously defined. The proposed metrics aim at highlighting the contribution of each portion of the spectrum with respect to the overall distortion of the signal. The rationale below those metrics consists of evaluating the “weight” of the distortion, in which 1 represents the maximum value, caused by each portion of the spectrum. This procedure provides system operators with a practical, high-level diagnostic tool to help with the quick identification of the root cause of power quality degradation. A note on the DC component: There is no proposed index considering the DC. This is because the measurement and subsequent mitigation/removal of such component is typically trivial, and it does not represent a challenge. A final metric that weighs the nature of the PQ degradation that is affecting the power network under analysis is defined as

(12) $$\rho =\frac{THD}{TWD}$$

In fact, a value equal to 1 for this index implies that the harmonics are the main cause of the deviation of the waveform from its ideal sinusoidal shape; consequently, other types of disturbances, such as interharmonics and noise, have a minimal contribution to the overall distortion of the measured signal.

Finally, the proposed indices and metrics, which will be implemented in the following section, are simple and flexible enough to be implemented in any computation system using data collected with any frequency resolution.

## 4. Validation by Simulation

### 4.1. Synthetic Signals

To validate the proposed indices and metrics, a test case was designed involving two synthetic signals, called S1 and S2, whose compositions are detailed in Table 1 and Table 2. Specifically, a 50 Hz signal was generated with a voltage of 15/√3 kV to emulate a medium-voltage (MV) system. Subsequently, three sets of harmonics were added as specified in Table 1, each corresponding to a particular range defined in the previous section (PQ, EX, and SH). The harmonic amplitudes, relative to the fundamental component, were chosen as 2.5%, 1%, and 0.25% for the three sets, respectively. These amplitude values were selected for simplicity and clarity, as they do not affect the validation of the indices. Signal S1 was further augmented with additional frequency components listed in Table 2, which include interharmonics within the PQ, EX, and SH ranges, as well as subharmonics. In contrast, signal S2 is identical to S1 but excludes the frequency components from Table 2. This distinction between S1 and S2—the first one encompassing a broader range of disturbances and the second one containing only harmonic components—ensures a more thorough validation of the proposed indices and metrics.

**Figure 1:**
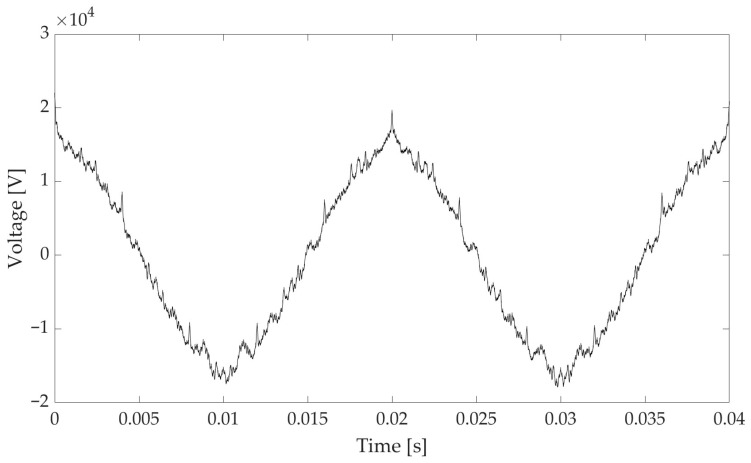
Time domain representation of signal S1.

The time domain representation of the signal S1 is depicted in Figure 1. Signal S2 is not plotted for the sake of brevity.

### 4.2. Results

To validate the indices described in Section 3, they are applied on signals S1 and S2, described above. In particular, the signal is assumed to be sampled with time windows of 1 s, 200 ms, and 20 ms to recreate the condition of a frequency resolution of 1 Hz, 5 Hz, and 50 Hz, respectively. Note that, in the considered case of a constant signal, there is no dynamicity in the frequency components. Therefore, the time window is solely used to change the frequency resolution and not for assessing specific time-varying frequency components. Finally, to obtain the frequency spectrum of the sampled signal in the time domain, the Fast Fourier Transform (FFT) is applied to each time window. Then, for the calculation of the proposed indices and metrics, only the amplitude values of the complex FFT are used. The phase information is not considered in this analysis, and the amplitude values are used for the calculation of the following indices (presented in Section 3).

The TWD of the two signals is listed in Table 3, which highlights the slight difference in the harmonic content between the two signals. According to the definition of the TWD, it is independent of the frequency resolution.

The THD, ${THD}_{PQ}$, ${THD}_{EX}$, ${THD}_{SH}$, and the ${THD}_{SUB}$ for signal S1, for each frequency resolution, are presented in Table 4. The first comment is on the ${THD}_{SUB}$, which does not have a value for the 50 Hz resolution for obvious reasons. Second, as expected, the higher the frequency resolution (i.e., smaller frequency steps), the higher the detail with which the frequency components can be analyzed. However, the most interesting comment is the third. In fact, focusing on a single-frequency resolution, it can be seen how the content of the signal can be judged at a glance. In the case of S1, the proposed indices provide a simple and clear indication of a significant distortion in the PQ frequency range, and a modest influence of high-frequency components.

Similarly, the results of the indices’ implementation for S2 are presented in Table 5. These results highlight the well-established principle that the absence of interharmonic components (including subharmonics) renders frequency resolution irrelevant to the signal’s frequency analysis. Nevertheless, in this simplified example, the proposed indices perform as effectively as they do with the more complex signal, S1.

A final comment on this first set of results is about the frequency resolution. It is known that the standard [5] suggests sampling the signal collecting 10 or 12 periods of the fundamental component (50 Hz or 60 Hz). However, it is not always possible due to the available instrumentation, duration of the signal, or the duration of the phenomena of interest. Therefore, the flexibility and simplicity of the indices become crucial for their widespread adoption.

Looking at the metrics $\rho_{PQ}$, $\rho_{EX}$, $\rho_{SH}$, and $\rho_{SUB}$, defined in Section 3, if applied to the results in Table 4 and Table 5, they provide the results presented in Table 6.

The metrics in Table 6 provide a clear information on the weight of each portion of the frequency range on the overall disturbance affecting the signal. Once again, the higher the frequency resolution, the better the analysis of the present harmonic components. It is interesting to highlight that, even in the case of a low-frequency resolution; for example, in the case of analysis of short duration phenomena, the metrics still provide a useful indication about the location of the disturbance.

A final note on the number of digits of the results: Table 3, Table 4, Table 5 and Table 6 contain values from simulations that, even if repeated, would not lead to different values. Therefore, the number of digits represented is a realistic value that could be possibly obtained in in-field conditions.

## 5. In-Field Validation

### 5.1. Scenario

After validating the proposed indices through a simulation, it is essential to evaluate their performance under more realistic conditions. The subsequent chapters detail the measurement setup, which comprises the electrical systems on which the tests are conducted to acquire the signals and apply the indices. Specifically, a previously characterized measurement setup was installed at the site of a PV plant installed at the Faculty of Engineering of the University of Bologna, particularly on the inverter used to inject power into the network. The output voltage of the inverter was measured, and the indices were subsequently calculated.

### 5.2. Measurement Setup and Power System Under Analysis

The system configuration of the electrical system under analysis consists of

- PV generation: The PV plant architecture contains 72 modules. Each module has a peak power rating of 400 Wp. Therefore, the nominal peak power of the PV system is equal to 28.8 kWp. The modules are electrically connected into seven strings.
- Power conditioning system: Power conversion and management are handled by two inverters—a 20-kW solar inverter processing the output from four strings and a 10-kW hybrid inverter processing the output from three other strings and a battery.
- Energy storage: An electrochemical battery system provides an energy capacity of 27.6 kWh.
- Loads: Mainly offices and classrooms.
- Grid.
- Monitoring and protective system: An electrical panel integrates circuit breakers, protective devices, and a smart meter for system monitoring and grid connection.

**Figure 2:**
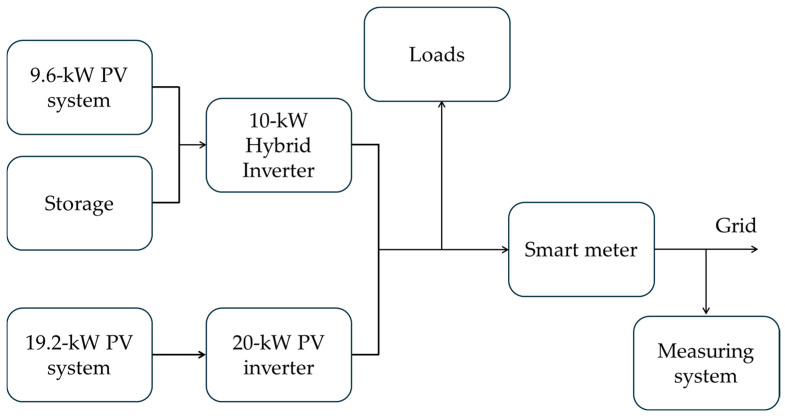
Block diagram of the power system under analysis and measurement setup.

With the help of Figure 2, the functioning of the system under analysis can be summarized as follows: the power generated by the PV is managed by two inverters and depending on the load demand, it is directly consumed by this load, stored in the battery or fed into the grid.

The measurement setup, used to acquire the inverter output voltage, consists of the following elements:

- A Hall effect sensor featuring (i) primary nominal rms voltage of 700 V, (ii) transformation ratio of 10, (iii) accuracy of 2%, (iv) frequency bandwidth from DC to 500 kHz, and (v) external power supply voltage required.
- NI DAQ 9222 data acquisition board featuring (i) ADC of 16 bits, analogue input voltage range of ±10 V, and a maximum sample rate of 500 kSa/s.
- Laptop that manages the automatic acquisition of voltage waveforms with dedicated running software.

The acquisition system is configured in the following way: every hour, a voltage waveform of 1 s duration is acquired at 500 kSa/s. The acquisition is automated by means of the Matlab 2024 software, and the acquired waveforms are saved in laptop memory. To better clarify what has been acquired, the reader can refer to Figure 2, in which it has been highlighted that the Hall effect sensor acquires the voltage that the inverter produces and that the final user would use.

In terms of repeatability, considering that acquisitions are performed on a real network, it is impossible to adopt the strategy of repeating the same measurements again. Therefore, the approach to quantify the accuracy and the goodness of the acquisitions is merely based on the contributions of the adopted instrumentation.

### 5.3. Evaluation of Results and Indices 

#### 5.3.1. Results from a Window Length of 1 s

This initial set of results is based on the full 1 s acquisition window, yielding a frequency resolution of 1 Hz. Although this resolution is not suitable for analyzing very fast events and does not align with standard requirements, it is used here as a reference case. The legacy and newly proposed indices for one of the acquisitions are presented in Table 7. For the same 1 s window, all indices were also computed at coarser frequency resolutions of 5 Hz and 50 Hz. As already completed when the simulation indices were calculated, the FFT is applied, and the amplitude values are used for the calculation of the PQ indices of Table 7 and Table 8. As shown, the frequency resolution significantly influences the outcomes for both the legacy and new indices. Notably, the new indices effectively emphasize the spectral components associated with signal distortion.

The indices presented in Table 7 for a single acquisition are plotted from Figure 3, Figure 4, Figure 5, Figure 6, Figure 7, Figure 8, Figure 9, Figure 10, Figure 11, Figure 12 and Figure 13 for the entire set of measurements.

**Figure 3:**
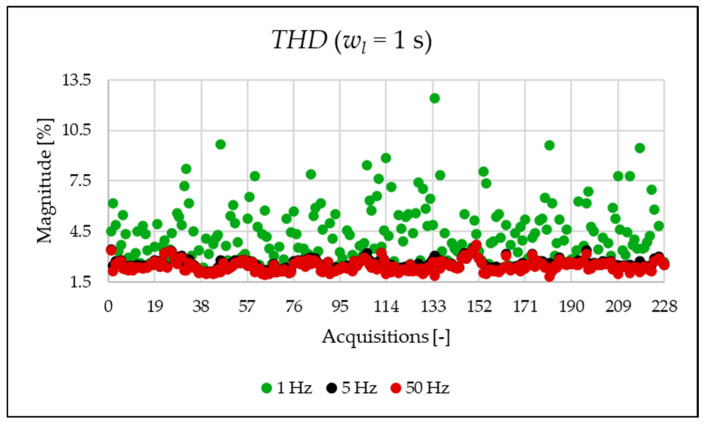
THD index of the 1 s time window.

**Figure 4:**
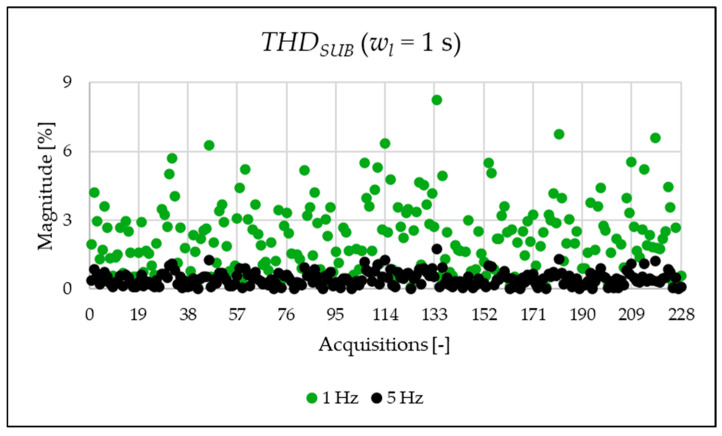
${THD}_{SUB}$ index of the 1 s time window.

In Figure 3 and Figure 4, the THD and the ${THD}_{SUB}$ are displayed. The results highlight a significant presence of subharmonic components when using a 1 Hz frequency resolution (see Figure 4 and Table 7). It is important to clarify that these components are not generated by the power system itself but are spectral leakage. The 1 s acquisition window is not perfectly synchronized with the 50 Hz power system frequency. As a result, the large energy of the fundamental component “leaks” into adjacent frequency bins, including those below 50 Hz. This effect is a well-known consequence of applying the FFT to non-synchronous signals with a rectangular window. As will be shown afterwards, this phenomenon is substantially reduced in the 20 ms window analysis, which is significantly more synchronized with respect to the fundamental. In conclusion, the main result from the two graphs is that, despite the leakage error that one operator might commit, the evaluation at different resolutions provides perspectives that do not emerge in other resolutions.

**Figure 5:**
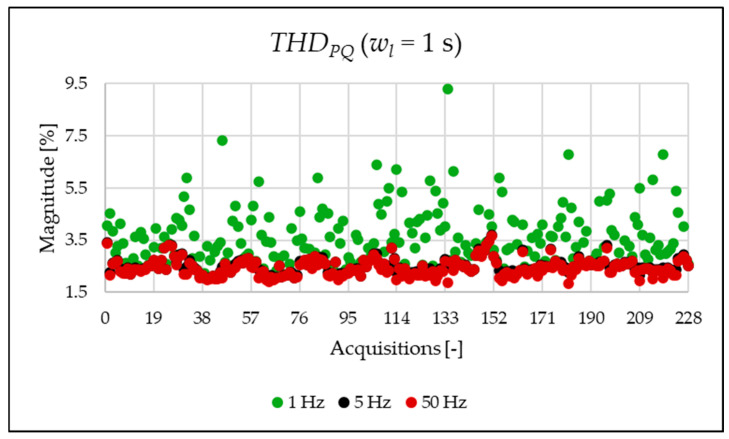
${THD}_{PQ}$ index of the 1 s time window.

**Figure 6:**
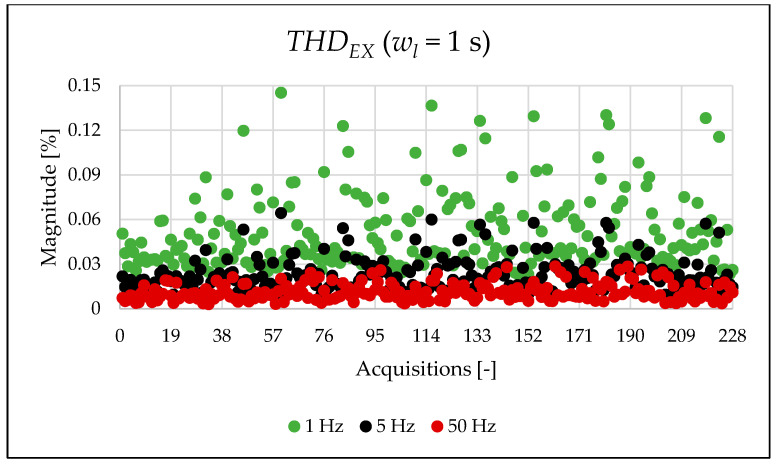
${THD}_{EX}$ index of the 1 s time window.

**Figure 7:**
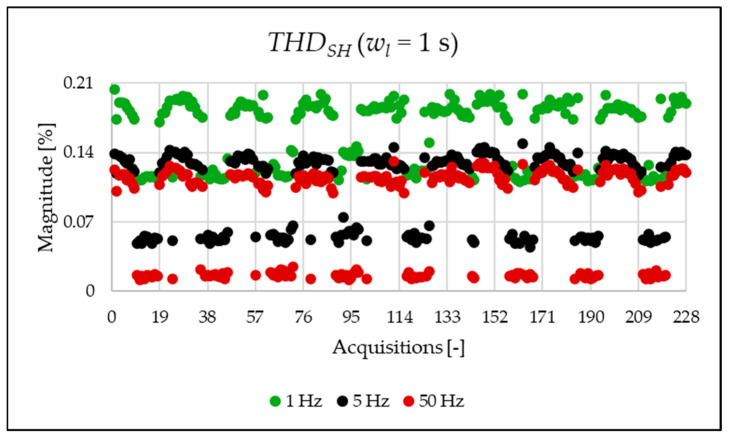
${THD}_{SH}$ index of the 1 s time window.

Figure 5, Figure 6 and Figure 7 present very interesting results about the signal content. In fact, the THD computed in the limited portion of the spectrum provides a first quantitative analysis of which components affect the signal. Within the case study, it can be observed how the PQ frequency range is the most populated one.

**Figure 8:**
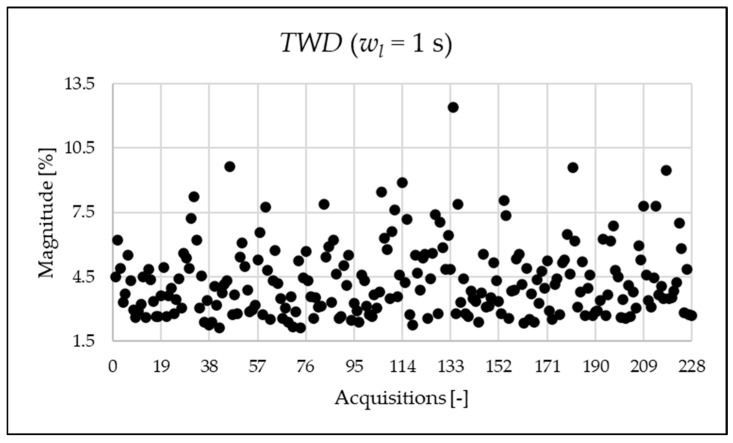
TWD index of the 1 s time window.

The TWD is depicted in Figure 8. It is useful when a detailed analysis of the frequency content of the signal is not needed.

**Figure 9:**
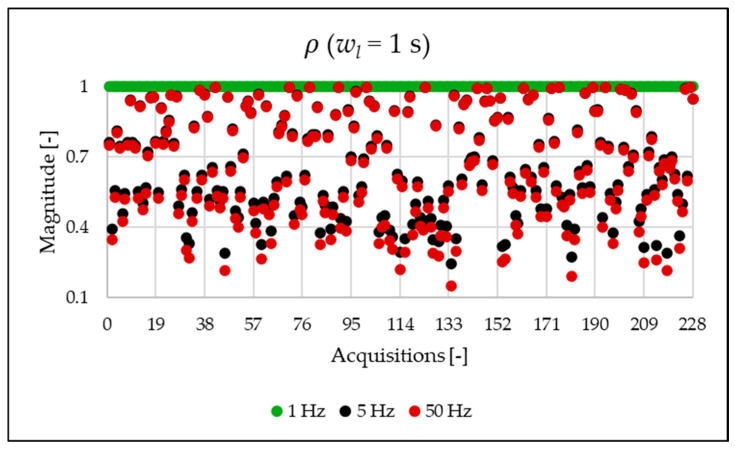
ρ index of the 1 s time window.

As already mentioned in Section 3, a ratio close to 1 between THD and TWD indicates a negligible presence of interharmonics. Therefore, the discrepancy observed in the analysis using a 1 Hz frequency versus 50 Hz in Figure 9 is expected and consistent with the nature of the corresponding index.

**Figure 10:**
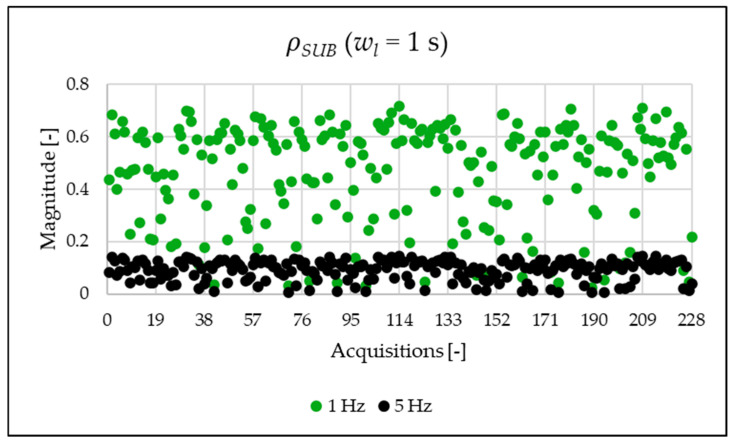
$\rho_{SUB}$ index of the 1 s time window.

**Figure 11:**
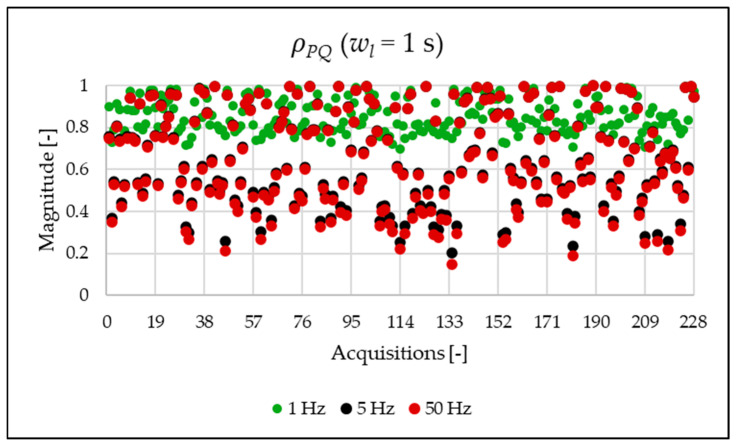
$\rho_{PQ}$ index of the 1 s time window.

**Figure 12:**
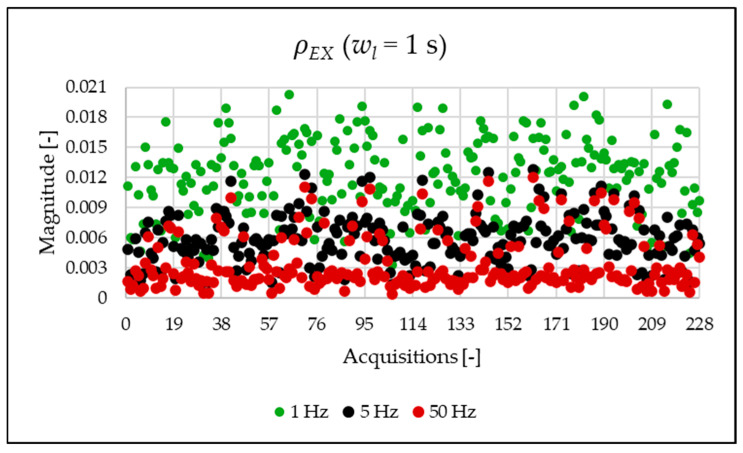
$\rho_{EX}$ index of the 1 s time window.

**Figure 13:**
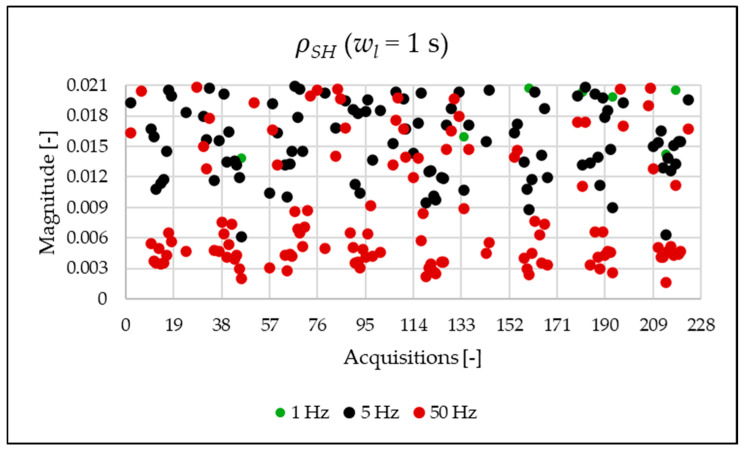
$\rho_{SH}$ index of the 1 s time window.

Figure 10, Figure 11, Figure 12 and Figure 13 show the proposed indices, each of which is dedicated to a specific portion of the spectrum. A first general comment is that, in most cases, the results obtained with a 50 Hz resolution are overlapping with those obtained with the 5 Hz resolution. This is always true but for the case of $\rho_{SH}$. This is reasonable because in that frequency range, the components are not related to the fundamental component anymore. A second comment is on the absolute values obtained. The proposed indices provide a clear and detailed indication about the significance of each portion of the spectrum. Such information is fundamental for an immediate quantitative PQ analysis of the signal.

#### 5.3.2. Results from a Window Length of 20 ms

In contrast to the previous analysis during which a 1 s acquisition window was used, this section evaluates the performance of these new indices using a 20-millisecond acquisition window. The choice of this shorter time interval offers two primary advantages for the calculation of the PQ indices: (i) mitigation of spectral leakage and (ii) reduced computational time. The first advantage yields indices that more accurately reflect the ‘true’ state of the electrical system under analysis and, consequently, the severity of the present harmonics. Concurrently, the reduced computational time facilitates a more rapid intervention due to the quicker availability of the index values. However, a frequency resolution limited to 50 Hz means that the effect of interharmonics is not incorporated into the index calculation. In conclusion, Table 8, similarly to Table 7, presents both the legacy and the new indices averaged for one acquisition. Each value of Table 8 is an average value calculated on fifty 20 ms time windows that make up each acquisition lasting 1 s.

The indices presented in Table 8 for a single acquisition are plotted from Figure 14, Figure 15, Figure 16, Figure 17, Figure 18, Figure 19, Figure 20, Figure 21 and Figure 22 for the entire set of measurements.

**Figure 14:**
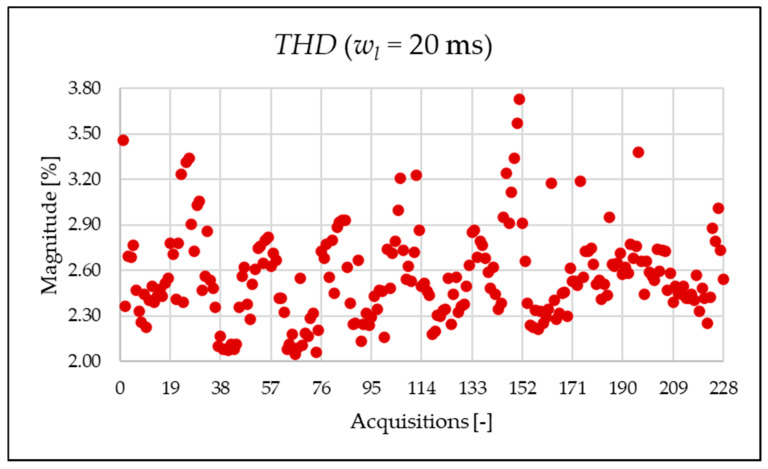
THD index of the 20 ms time window.

**Figure 15:**
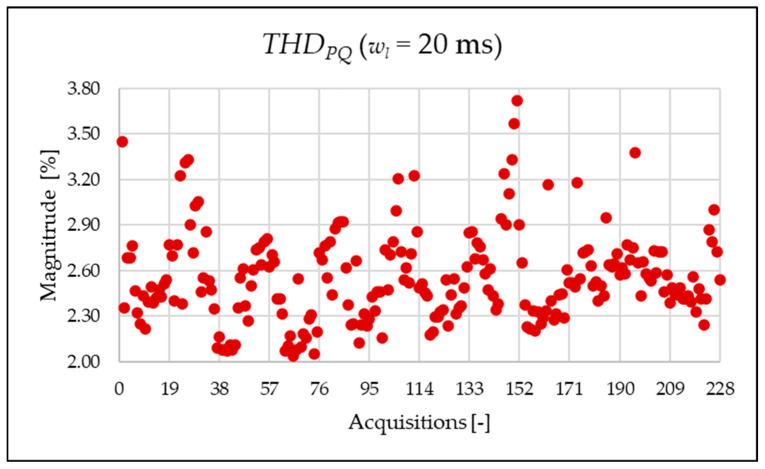
${THD}_{PQ}$ index of the 20 ms time window.

**Figure 16:**
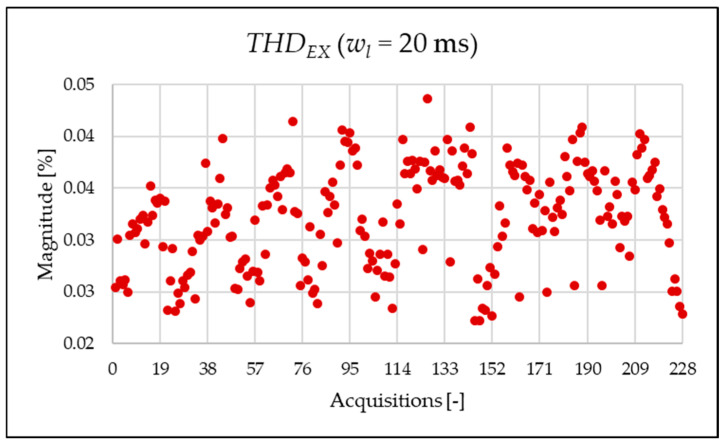
${THD}_{EX}$ index of the 20 ms time window.

**Figure 17:**
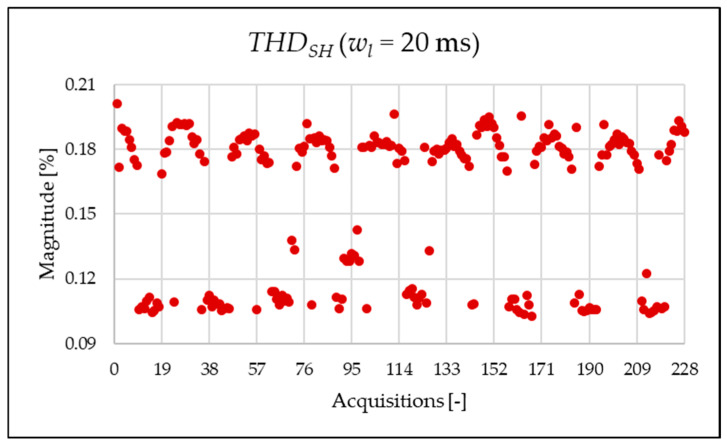
${THD}_{SH}$ index of the 20 ms time window.

Figure 14, Figure 15, Figure 16 and Figure 17 represent the THD, ${THD}_{PQ}$, ${THD}_{EX}$ and ${THD}_{SH}$, respectively. As expected from the use of a shorter analysis window, the indices displayed a general decline in value due to a lower presence of leakage. Even in this case of a shorter window, the PQ band is the predominant one and causes the greatest harmonic pollution of the network. However, the presence of both static converters and a storage system leads to a clear presence of high-frequency harmonics in the supraharmonic (SH) frequency range, which exhibit fluctuating behavior throughout the day.

**Figure 18:**
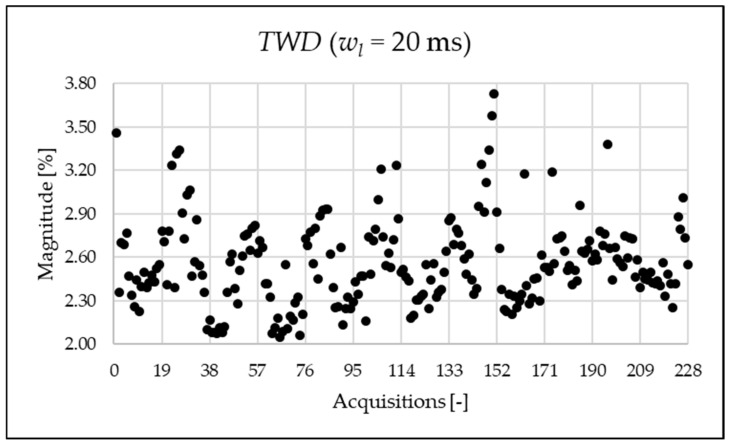
TWD index of the 20 ms time window.

**Figure 19:**
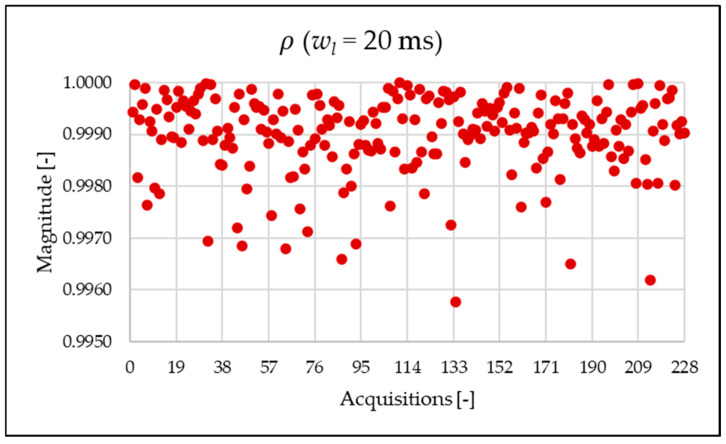
ρ index of the 20 ms time window.

From Figure 19, the metric ρ is almost unitary. The reason is clear when comparing the THD and TWD indices, represented in Figure 14 and Figure 18, respectively, which are very similar in value.

**Figure 20:**
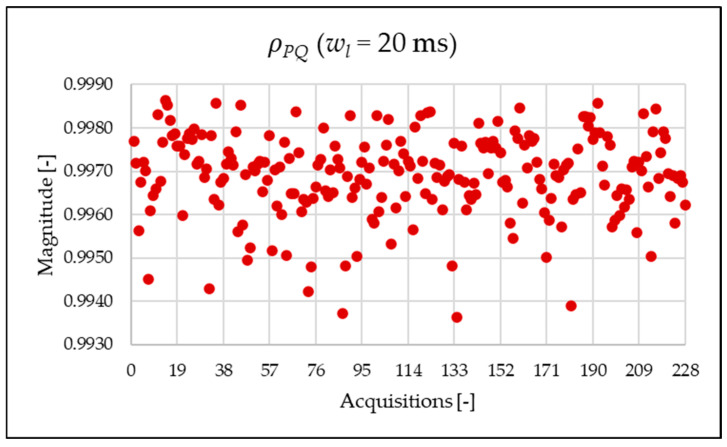
$\rho_{PQ}$ index of the 20 ms time window.

**Figure 21:**
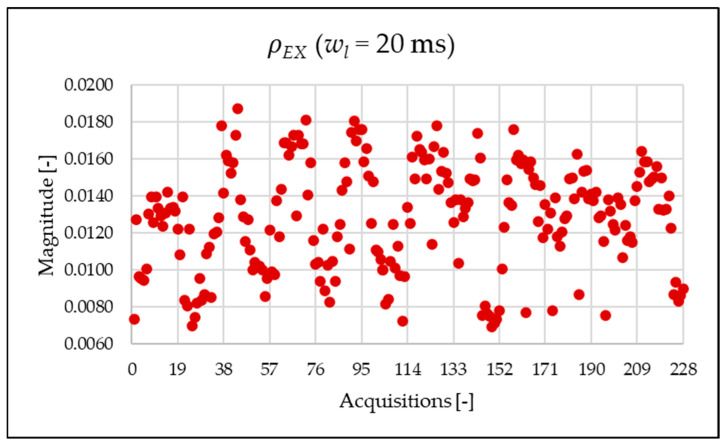
$\rho_{EX}$ index of the 20 ms time window.

**Figure 22:**
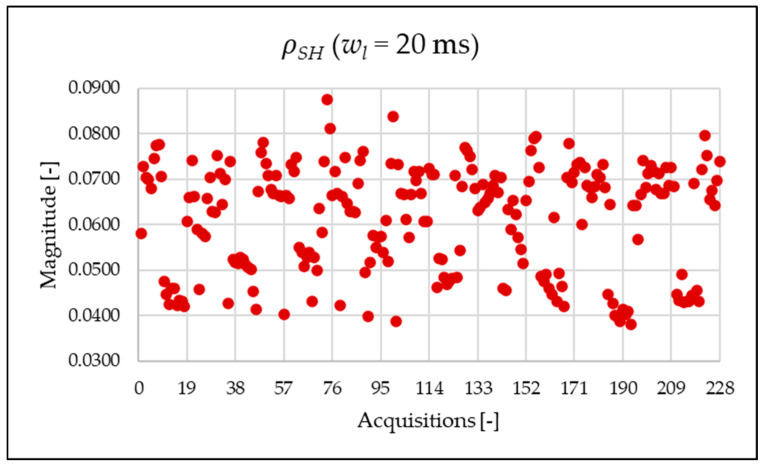
$\rho_{SH}$ index of the 20 ms time window.

Similarly to what is seen in Figure 11, Figure 12 and Figure 13, Figure 20, Figure 21 and Figure 22 show the proposed indices for each selected frequency band in the case where the signal is analyzed over a 20 ms time window. Again, the PQ band, as expected, is the band that contributes most to harmonic pollution of the network compared to the other two bands (EX and SH). In conclusion, the proposed indices offer a clear and detailed insight into the significance of each part of the spectrum. Furthermore, thanks to their modular nature, these new indices enable users to analyze their network over customizable frequency ranges—for example, based on the converter technology used and its corresponding switching frequency.

After presenting all results, a comment on their variability and accuracy is needed. First, it is commonly difficult to perform repeated measurements on real networks due to lack of stability of the signal. Therefore, the statistical approach to reduce uncertainty cannot be used. Second, in this case, the information that is received from the instrumentation is crucial. In fact, as presented in [30], the authors guide the user to the calculation of the uncertainty associated with THD indices with a closed-form expression that uses the collected waveforms as a starting point.

As a final note, accurate signal analysis depends on many factors—such as the acquisition window, resolution, windowing technique, filters, and instrumentation. However, the approach presented in this research can be applied independently of these aspects. In other words, it is flexible enough to adapt to all such variables, which makes it particularly valuable for real in-field applications.

## 6. Conclusions

This paper introduces a set of simple yet effective power quality (PQ) indices aimed at improving the evaluation of harmonic distortions across a wide frequency range (1 Hz–150 kHz). Addressing current gaps in methodologies and standards, the proposed indices are flexible and robust, capturing key characteristics of power disturbances independently of acquisition conditions. Their performance is demonstrated through validation on both synthetic and real signals, confirming their capability to identify and quantify contributions from distinct spectral components, including harmonics, interharmonics, and subharmonics. Beyond simplifying PQ analysis, these indices support practical decision-making for system operators by pinpointing the frequency ranges that most significantly contribute to signal distortion. Their straightforward implementation also ensures adaptability to diverse measurement systems and frequency resolutions, making them suitable for widespread adoption. Collectively, these results advance power quality monitoring and mitigation strategies, particularly in modern electrical systems marked by renewable-energy integration and the proliferation of non-linear loads.

## Figures and Tables

**Table 1:** Harmonic content of signal S1 and S2.

Range [-]	Frequency [Hz]	% of Fundamental
PQ	150	2.5
	250	2.5
	350	2.5
	450	2.5
	550	2.5
	800	2.5
	1000	2.5
	1250	2.5
	2000	2.5
	2500	2.5
EX	3000	1
	3750	1
	4500	1
	5000	1
	5750	1
	6250	1
	7000	1
	7750	1
	8300	1
	9000	1
SH	22,500	0.25
	37,500	0.25
	50,000	0.25
	62,500	0.25
	75,000	0.25
	100,000	0.25
	112,500	0.25
	125,000	0.25
	137,500	0.25
	150,000	0.25

**Table 2:** Interharmonic content of signal S1.

Range [-]	Frequency [Hz]	% of Fundamental
SUB	17	1.25
	25	1.25
PQ	720	1.25
	1329	1.25
	2275	1.25
EX	2835	0.5
	4259	0.5
	6120	0.5
	7330	0.5
	8740	0.5
SH	10,210	0.125
	50,020	0.125
	90,029	0.125
	120,060	0.125
	147,480	0.125

**Table 3:** TWD of signals S1 and S2.

Index [%]	S1	S2
TWD	9.07	8.55

**Table 4:** Different THD indices results, for each frequency resolution, for S1.

Index [%]	Frequency Resolution [Hz]		
	50	5	1
THD	8.55	8.96	9.07
${THD}_{PQ}$	7.91	8.10	8.20
${THD}_{EX}$	3.16	3.31	3.35
${THD}_{SH}$	0.79	0.83	0.84
${THD}_{SUB}$	-	1.25	1.77

**Table 5:** Different THD indices results, for each frequency resolution, for S2.

Index [%]	Frequency Resolution [Hz]		
	50	5	1
THD	8.55	8.55	8.55
${THD}_{PQ}$	7.91	7.91	7.91
${THD}_{EX}$	3.16	3.16	3.16
${THD}_{SH}$	0.79	0.79	0.79
${THD}_{SUB}$	-	-	-

**Table 6:** Application of the new metrics to signals S1 and S2.

Index [-]	Signal [-]	Frequency Resolution [Hz]		
		50	5	1
	S1	0.943	0.989	1
	S2	1	1	1
$\rho_{PQ}$	S1	0.871	0.893	0.903
	S2	0.924	0.924	0.924
$\rho_{EX}$	S1	0.348	0.365	0.369
	S2	0.369	0.369	0.369
$\rho_{SH}$	S1	0.087	0.091	0.092
	S2	0.092	0.092	0.092
$\rho_{SUB}$	S1	-	0.137	0.194
	S2	-	-	-

**Table 7:** PQ index vs. frequency resolutions (window length 1 s).

		Frequency Resolution [Hz]		
		50	5	1
Index [%]	THD	2.33	2.53	5.49
	${THD}_{SUB}$	*-*	0.74	3.61
	${THD}_{PQ}$	2.32	2.41	4.13
	${THD}_{EX}$	0.004	0.009	0.026
	${THD}_{SH}$	0.11	0.13	0.19
	TWD	5.49		
Index [-]	ρ	0.42	0.46	1
	$\rho_{SUB}$	*-*	0.14	0.66
	$\rho_{PQ}$	0.42	0.44	0.75
	$\rho_{EX}$	0.0007	0.0017	0.0047
	$\rho_{SH}$	0.020	0.024	0.034

**Table 8:** PQ index vs. frequency resolutions (window length 20 ms).

		Frequency Resolution [Hz]
		50
Index [%]	THD	2.33
	${THD}_{PQ}$	2.32
	${THD}_{EX}$	0.03
	${THD}_{SH}$	0.18
	TWD	2.34
Index [-]	ρ	0.9976
	$\rho_{PQ}$	0.9945
	$\rho_{EX}$	0.0130
	$\rho_{SH}$	0.1808

## Data Availability

Data is unavailable due to privacy.
